# Platelet Effects of Anti-diabetic Therapies: New Perspectives in the Management of Patients with Diabetes and Cardiovascular Disease

**DOI:** 10.3389/fphar.2021.670155

**Published:** 2021-05-12

**Authors:** Annunziata Nusca, Dario Tuccinardi, Silvia Pieralice, Sara Giannone, Myriam Carpenito, Lavinia Monte, Mikiko Watanabe, Ilaria Cavallari, Ernesto Maddaloni, Gian Paolo Ussia, Silvia Manfrini, Francesco Grigioni

**Affiliations:** ^1^ Unit of Cardiac Sciences, Department of Medicine, Campus Bio-Medico University of Rome, Rome, Italy; ^2^ Unit of Endocrinology and Diabetes, Department of Medicine, Campus Bio-Medico University of Rome, Rome, Italy; ^3^ Department of Experimental Medicine, Section of Medical Pathophysiology, Food Science and Endocrinology, Sapienza University of Rome, Rome, Italy; ^4^ Department of Experimental Medicine, Sapienza University of Rome, Rome, Italy

**Keywords:** anti-diabetic therapies, glucose-lowering drugs, platelet, anti-platelet agents, cardiovascular disease, thrombotic risk, diabetes

## Abstract

In type 2 diabetes, anti-thrombotic management is challenging, and current anti-platelet agents have demonstrated reduced efficacy. Old and new anti-diabetic drugs exhibited—besides lowering blood glucose levels—direct and indirect effects on platelet function and on thrombotic milieu, eventually conditioning cardiovascular outcomes. The present review summarizes existing evidence on the effects of glucose-lowering agents on platelet properties, addressing pre-clinical and clinical research, as well as drug–drug interactions with anti-platelet agents. We aimed at expanding clinicians’ understanding by highlighting new opportunities for an optimal management of patients with diabetes and cardiovascular disease. We suggest how an improvement of the thrombotic risk in this large population of patients may be achieved by a careful and tailored combination of anti-diabetic and anti-platelet therapies.

## Introduction

Type 2 Diabetes (T2D) represents a critical risk factor for cardiovascular disease (CVD). The incidence of acute thrombotic events such as acute myocardial infarction (MI) and stroke is significantly greater in diabetic patients compared to non-diabetics (Tancredi et al., 2015; Cavender et al., 2015; Nusca et al., 2018). Similarly, T2D is associated with worse prognosis following MI, increasing the risk of heart failure (HF) and mortality on long-term follow-up (Murcia et al., 2004).

Platelet dysfunction plays a central role in building up the cardiovascular risk of diabetic patients, since T2D is characterized by an enhanced thrombotic state related to endothelial dysfunction, activation of the coagulation cascade and increased platelet reactivity (Murcia et al., 2004; Gæde et al., 2003; Gresele et al., 2010; Nusca et al., 2019).

The pathophysiology underlying such abnormal platelet response has been largely investigated. Platelets from diabetic patients show high oxidative stress levels due to increased production of reactive oxygen species (ROS), partly mediated by activation of NADPH oxidase 2, in turn leading to amplified aggregation (Cangemi et al., 2012; Carnevale et al., 2014). T2D is associated with greater production of 8-iso-prostaglandin (PG) F2α, a stable compound of non-cyclooxygenase peroxidation of arachidonic acid inducing vasoconstriction and platelet activation (Davì et al., 1999). Furthermore, an enhanced thromboxane (TXB) platelet biosynthesis was demonstrated in diabetic patients (Davì et al., 1990), together with increased platelet susceptibility to aggregating agonists [adenosine diphosphate (ADP), thrombin] and diminished sensitivity to anti-thrombotic molecules such as prostacyclin (PGI2) and nitric oxide (NO) (Vinik et al., 2001). Finally, hyperglycemia has been reported to significantly affect the expression of several platelet and circulating microRNAs in diabetic patients; these small, non-coding RNA molecules have a central role in the modulation of platelet activity and represent potential biomarkers to investigate the response to specific anti-platelet treatments (Carino et al., 2016; Pordzik et al., 2019). Notably, increased shear stress in diseased vessels of diabetic patients may trigger sustained platelet aggregation independent of soluble agonists as well (Nesbitt et al., 2009), a particularly interesting aspect since major antiplatelet agents act exactly by preventing the release of these agonists.

According to this evidence, platelet hyper-reactivity certainly concurs to the development of life-threatening thrombotic complications in T2D patients, with current antiplatelet agents proven to be less effective in diabetic patients, with a high percentage of low or non-responders (Geisler et al., 2007; Angiolillo et al., 2014; Rivas Rios et al., 2018). Of note, an impaired response to antiplatelet therapy has been associated with worse long-term clinical outcomes in several investigations (Angiolillo et al., 2007; Patti et al., 2008; Brar et al., 2011). Altogether, this accounts for the continuous efforts to improve anti-platelet strategy in diabetic patients with CVD, especially when subjected to percutaneous coronary interventions (PCI) (Angiolillo and Ueno, 2011). More effective P2Y12 receptor antagonists such as ticagrelor and prasugrel have been associated with reduced thrombotic events in this high-risk subgroup, although this comes at the cost of higher hemorrhagic risk (Wiviott et al., 2008; James et al., 2010).

**FIGURE 1:**
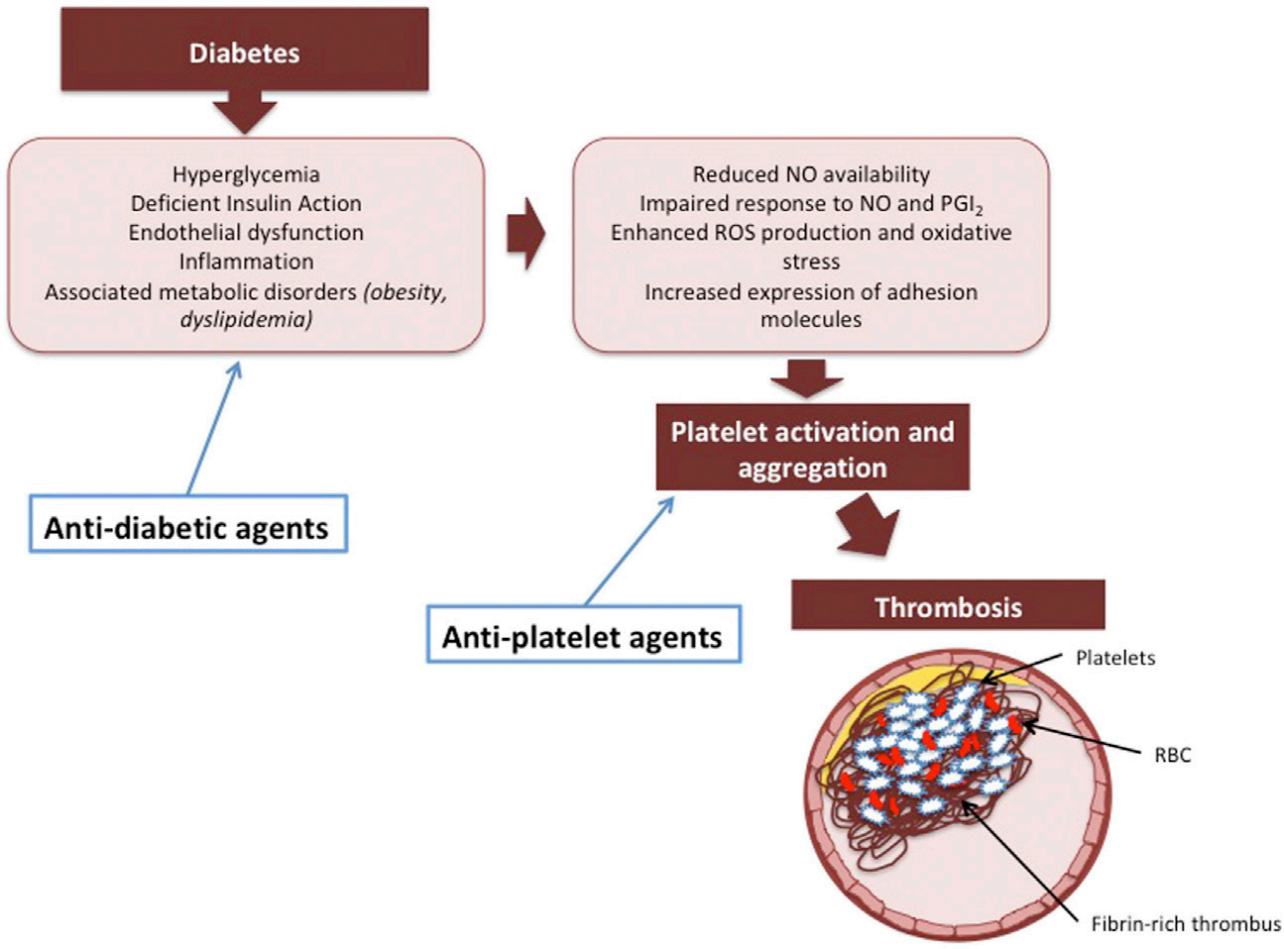
The potential synergistic effect of anti-diabetic and anti-platelet agents on preventing thrombosis in diabetic patients.

Nowadays, a new paradigm shift in diabetes treatment occurs, since clear evidence of cardiovascular benefits associated with novel anti-diabetes drugs has been reported in several randomized trials, moving toward a less “glucocentric” and more “cardioprotective” standpoint (Zinman et al., 2015; Neal et al., 2017; Wiviott et al., 2019; Marso, Daniels, et al., 2016; Kristensen et al., 2019; Marso, Bain, et al., 2016; Holman R. R. et al., 2017). Some glucose-lowering agents have also demonstrated antithrombotic effects in observational studies. The potential benefit on platelets may be related to the normalization of glycemic control, but other additional direct antithrombotic and anti-inflammatory mechanisms may be involved. These findings are clinically relevant since the modulation of platelet activation by anti-diabetic drugs may mitigate the risk of thrombotic events and contribute to cardiovascular protection in diabetic patients (Figure 1).

Nonetheless, the impact of “old and new” glucose-lowering agents on platelet function has not been completely clarified yet. Moreover, studies investigating insulin in this setting are limited and controversial. Herein, we review and discuss current knowledge about the impact of anti-diabetic agents on platelet function and their possible implications for T2D and CVD management.

## Metformin

Metformin is a biguanide and exerts its antidiabetic action as an insulin sensitizer by inhibiting gluconeogenesis and stimulating muscle tissue and other insulin-dependent tissues to use glucose (Bailey, 1992). It also determines a lower absorption of glucose in the gastrointestinal tract. Of note, metformin does not induce hypoglycemia, and aside from gastrointestinal side effects when at a higher dosage, it represents a well-tolerated drug (Zilov et al., 2019). The American Diabetes Association recommends metformin as first-line therapy for T2D in newly diagnosed cases and it is the most widely used anti-diabetic drug.

### Cardiovascular Outcomes

Metformin is associated with decreased cardiovascular risk in long-term clinical trials. In the UK Prospective Diabetes Study Group (UKPDS), metformin showed a significant reduction in cardiovascular events (MI and stroke) or all-cause mortality compared with diet or chlorpropamide or insulin (King et al., 1999). Similar findings were reported in the Hyperinsulinemia: the Outcome of its Metabolic Effects (HOME) randomized trial; in this study, treatment with metformin improved glycemic control and reduced macrovascular complications (MI, stroke, peripheral vascular disease) in diabetic patients already on insulin therapy on a 4.3 years clinical follow-up (Kooy et al., 2009). Of note, the HOME trial was largely conducted in patients with T2D without previous CVD. Interestingly, a longer duration of metformin treatment seems to improve survival, whereas, adding sulphonylureas to metformin has been demonstrated to attenuate its cardiovascular benefit (Lamanna et al., 2011). Table 1 summarizes the cardiovascular effects of metformin as well as those of all other anti-diabetic agents discussed in this review.

**TABLE 1:** Cardiovascular and platelet effects of anti-diabetic agents.

Molecule	Glucose lowering mechanism	Clinical cardiovascular effects	Platelet effects
Metformin	↓ Hepatic gluconeogenesis	↓ Cardiovascular endpoints (MI and stroke) and all-cause mortality (UKPDS) (King et al., 1999)	*Preclinical*
	↑ Muscle tissue and other insulin-dependent tissues glucose uptake	↓ Macrovascular complications (MI, stroke, peripheral vascular disease) (HOME) (Kooy et al., 2009)	↓ ADP, collagen and arachidonic acid-induced platelet aggregation (Tremoli et al., 1982; Gin et al., 1989)
	↓ GI absorption of glucose		↓ Production of superoxide anion (O2-) (Gargiulo et al., 2002)
			↓ PLT activation and extracellular mitochondrial DNA release
			*Clinical*
			↓ 11-dhTXB2 urinary excretion (Formoso et al., 2008)
			↓ 8-iso-pg F2α excretion (Formoso et al., 2008)
			↓ Mean PLT volume (Dolasik et al., 2013)
Sulphonylureas	↑ Insulin secretion (through pancreatic beta cells receptors)	↓ Cardiovascular benefit vs metformin alone (UKPDS) (King et al., 1999)	*Preclinical*
	↑ Number of peripheral insulin receptors (through extra-pancreatic receptors)	↑ Risk of cardiovascular hospitalization/mortality (Rao et al., 2008)	↓ ADP-induced platelet aggregation
	↑ Glucose intake by tissues	↑ Risk of stroke and overall mortality, no effect on MACEs (Monami et al., 2013)	↓ PLT adhesiveness (Klaff et al., 1981; Satoh et al., 1994)
	↓ Hepatic glucose production	Neutral effect with regard of all-cause mortality, cardiovascular mortality, MI or stroke with second or third-generation molecules (varvaki Rados et al., 2016)	↓ Oxidative stress (Klaff et al., 1981; Violi et al., 1982)
			↓ Cyclooxygenase and lipoxygenase pathways (Satoh et al., 1994)
			*Clinical*
			↓ PLT aggregation (de Bellis et al., 1984; Jennings et al., 1992; Konya et al., 2010)
Thiazolidinediones	↓ Circulating fatty acids promoting ability to store lipids	Pioglitazone	*Preclinical*
	↓ Insulin resistance	Neutral effect on major cardiovascular end points (PROactive, Dormandy et al., 2005); ↓stroke or MI (IRIS) (Viscoli et al., 2014)	↓ ADP-induced PLT aggregation (Li et al., 2005)
	↑ Insulin sensitivity and glucose uptake in muscle	Rosiglitazone	↓ P selectin levels (Bodary et al., 2005)
		↑ risk of cardiovascular events (Selvin et al., 2008; Home et al., 2009); no increase in the incidence of MI or cardiovascular death; ↑ heart failure hospitalizations (RECORD) (Home et al., 2007; Home et al., 2009)	*Clinical*
			↓ Inflammation and macrophage recruitment (Yau et al., 2013)
			↓ E selectin (Hetzel et al., 2005), vWillebrand, SCD40L (Sidhu et al., 2003; schöndorf T. et al., 2011), PAI-1 (Derosa et al., 2005)
			↓ 11-dhTXB2 (Basili et al., 2006)
Acarbose	↓ α-glucosidase in the intestinal tract	↓ Risk cardiovascular events (STOP-NIDDM) (Chiasson et al., 2003)	*Preclinical*
	↓ Salivary α-glucosidase	↓ Progression of carotid intima-media thickness (markolf Hanefeld et al., 2004)	↓ PLT-bound fibrinogen and ↓ P selectin platelet exposure (Schäfer et al., 2004)
	↓ Pancreatic α-amylase		↓ Platelet-monocyte aggregates formation (Kaplar et al., 2001)
	↑ GLP-1		*Clinical*
			↓ PLT activation and oxidative stress markers (Santilli et al., 2010)
Dipeptidyl peptidase-4 inhibitors	↓ Breakdown GLP-1 and GIP	No clear benefit on MACEs incidence vs placebo (TECOS/EXAMINE/CAROLINA/TIMI) (White et al., 2013; Green et al., 2015; Marx et al., 2015; Udell et al., 2015)	*Preclinical*
			↑ cAMP formation and PKA activation (Steven et al., 2017)
			↓ Plasma fibrinogen and PAI-1 (Dardik et al., 2003)
			↓ Soluble levels of CD40 (Mason et al., 2011)
			↓ Inflammatory and thrombogenic gene expression (Maeda et al., 2012; Birnbaum et al., 2016)
			↓ Platelet mitochondrial respiration and platelet aggregation (Li et al., 2020)
			*Clinical*
			↓ Intracellular free calcium and tyrosine phosphorylation→ ↓ PLT aggregation (Gupta et al., 2012)
Sodium–glucose cotransporter 2 inhibitors	↓ Kidney sodium glucose cotransporters → urinary net loss of sodium and glucose	↓ Incidence of MACE, cardiovascular death and hospitalization for HF (EMPA-REG OUTCOME, Zinman et al., 2015; CANVAS program, Neal et al., 2017, and DECLARE-TIMI 58, Wiviott et al., 2018)	*Preclinical*
			↓ ADP- induced PLT activation (Spigoni et al., 2020)
			↓ P selectin mRNA expression (Steven et al., 2017)
			↓ ROS and ↑ NO bioavailability (Smyth et al., 2017)
			↓ Advanced glycation end products, ↑ eNOS activation and ↓ interstitial and periarterial NO stress (Aroor et al., 2018)
GLP-1 receptor agonists	↑ Insulin secretion and ↓ glucagon in a glucose-dependent manner	↓ MACEs and ↓ fatal and non-fatal MI (ELIXA, LEADER, SUSTAIN- 6, EXSCEL, harmony outcomes, REWIND and PIONEER 6 (Kristensen et al., 2019)	*Preclinical*
	↓ Beta-cell apoptosis		↓ Thrombin-, ADP-, and collagen-induced PLT aggregation mediated by cAMP-induced PKA activation and increased eNOS enzymatic activity (Cameron-Vendrig et al., 2016; Steven et al., 2017)
	↑ Beta-cell neogenesis		↓ ROS production (Oeseburg et al., 2010; Ceriello et al., 2013)
	↓ Circulating lipoproteins		*Clinical*
	↓ Gastric emptying		↑ cGMP production and ↑ VASP-ser239 phosphorylation and ↓ PI3-K/Akt and MAPK/erk-2 pathways → ↑ NO bioavailability and ↓ ROS production (Barale et al., 2017)
	↑ Satiety		↓ Platelet P-selectin expression (Kahal et al., 2015)

ADP, adenosine disphosphate; ERK, extracellular signal-regulated kinases; eNOS, endothelial nitric oxide synthase; GI, gastro intestinal; GIP, gastric inhibitory peptide; GLP-1, glucagon like peptide-1; HF, heart failure; MACEs, major adverse cardiac events; MAPK, mitogen-activated protein kinases; MI, myocardial infarction; NO, nitric oxide; PAI-1, plasminogen activator inhibitor-1; PG, prostaglandin; PI3K, phosphatidyl inositol-3 kinase; PKA, protein kinase A; PLT, platelet; ROS, reactive oxygen species; TXB, thromboxane; VASP, vasodilator-stimulated phosphoprotein.

### Effects on Platelets

#### Preclinical Studies

In an experimental study, metformin was able to normalize platelet response to different stimuli such as collagen and arachidonic acid in a hypercholesterolemic rabbit model (Tremoli et al., 1982). Moreover, metformin has been demonstrated to prevent venous and arterial thrombosis by inhibiting platelet activation and extracellular mitochondrial DNA release in carotid and vena cava animal models (Xin et al., 2016).

#### Clinical Studies

One of the first *in vivo* studies investigating the potential effect of metformin on platelets showed a significant decrease in maximum ADP-induced platelet aggregation with this agent (Gin et al., 1989). Metformin, administered with a dose of 1,700 mg daily, was added on top of insulin therapy in well-controlled type 1 diabetes (T1D) patients. After 21 days of treatment, no appreciable changes occurred on metabolic factors, such as fibrinogen, cholesterol and triglycerides levels, HbA1c and mean glycemia levels; nevertheless, the maximum platelet aggregation induced by ADP (1.25, 2.5, and 5 μmol doses) decreased significantly (Gin et al., 1989). Another small study in newly diagnosed T2D patients found that metformin improved oxidative stress, preserved anti-oxidant function and decreased platelet activation (Formoso et al., 2008). Specifically, metformin, but not gliclazide treatment, was associated with a significant decrease in 11-dehydro-TXB2 (11-dhTXB2) urinary excretion, a reliable parameter for the measurement of platelet activation *in vivo* (Formoso et al., 2008). Moreover, metformin has been demonstrated to significantly reduce 8-iso-PG F2α excretion after 12 weeks of treatment, leading to a concurrent increase in anti-oxidant vitamin A and E concentrations (Formoso et al., 2008). A reduced production of platelet superoxide anion (O2-) was also reported in patients treated with metformin compared with glibenclamide or diet (Gargiulo et al., 2002). Finally, long-term metformin seemed to influence dimensional platelet parameters: Dolasık et al. (2013) reported a decreased mean platelet volume in diabetic patients on metformin. Despite what has been described so far, Collier et al. suggested that the benefit of metformin on platelet reactivity was not a class effect but instead related to the glycemic control optimization. In fact, they reported no difference in terms of platelet parameters between metformin and gliclazide when an optimal glucose control was achieved (Collier et al., 1989). The main platelet effects of metformin are reported in Table 1, among the platelet effects of all other anti-diabetic molecules evaluated in this review.

#### Interaction with Anti-Platelet Drugs

Few studies also investigated the interaction between metformin and anti-platelet drugs, such as aspirin and clopidogrel. The use of metformin among diabetic patients on aspirin therapy was associated with a significantly reduced platelet activation: a higher percentage of patients on metformin in addition to aspirin presented a reduction of 11-dhTXB2 above 75% compared with those on aspirin only (52 vs. 20%, *p* = 0.027) (Gonçalves et al., 2014). Conversely, another recent study investigated the impact of metformin use on platelet reactivity assessed at 30–90 days by multiple-electrode aggregometry in T2D patients treated with dual antiplatelet therapy (DAPT) after PCI, and no apparent association between the use of metformin and platelet reactivity was found (Verdoia et al., 2021). Table 2 shows drug-drug interactions between anti-diabetic and anti-platelet agents with potential molecular mechanisms.

**TABLE 2:** Drug–drug interactions between anti-diabetic and anti-platelet agents.

Molecule	Interaction			Evidence and possible mechanisms
	ASA	ASA + clopidogrel	Clopidogrel	
Metformin	+	±	n/a	↓ 11-dhTXB2 in addition to aspirin vs. aspirin only (Gonçalves et al., 2014); No effect on platelet reactivity in patients treated with metformin and DAPT (Verdoia et al., 2021)
Sulphonylureas	n/a	–	n/a	↑ Risk of high on-clopidogrel platelet reactivity due to competition for isoenzyme CYP2C9 between sulfonylureas and clopidogrel (Harmsze et al., 2011)
Thiazolidinediones	+	n/a	+/-	Pioglitazone potentiates aspirin-induced platelet inhibition (Mogan et al., 2012) No enhancement in clopidogrel anti-platelet effect with TDZs (Suryadevara et al., 2012); on the other side, clopidogrel may increase the exposure to pioglitazone effects by inhibiting its CYP2C8-mediated biotransformation (Itkonen et al., 2016)
Acarbose	n/a	n/a	n/a	n/a
Dipeptidyl peptidase-4 inhibitors	n/a	n/a	n/a	n/a
Sodium–glucose cotransporter 2 inhibitors	n/a	n/a	n/a	n/a
GLP-1 receptor agonists	n/a	n/a	n/a	n/a

(+) synergic effect (±) neutral effect (−) antagonistic effect (n/a) not applicable. ASA, acetylsalicylic acid; DAPT, dual antiplatelet therapy; TXB, thromboxane; TZDs, thiazolidinediones.

## Sulphonylureas

Sulphonylureas can be divided into first (chlorpropamide) and second-generation (e.g., glibenclamide, gliclazide, glipizide) molecules. This class of agents stimulates insulin secretion by binding to receptors on pancreatic beta cells; furthermore, they have extra-pancreatic actions, inducing an increase in the number of peripheral insulin receptors, thus raising glucose uptake and inhibiting hepatic gluconeogenesis (Proks et al., 2002).

### Cardiovascular Outcomes

Previous studies did not demonstrate any cardiovascular benefit of sulphonylureas but rather raised concerns regarding their safety (Table 1). In fact, experimental data suggested that these agents might impair myocardiocytes function by interfering with a myocardial ATP-sensitive potassium channel (Scognamiglio et al., 2002). Furthermore, hypoglycemia is more frequent with sulphonylureas than metformin (Maruthur et al., 2016).

Accordingly, in the previously reported UKPDS trial, adding sulphonylureas to metformin resulted in a reduced cardiovascular improvement (King et al., 1999). Likewise, other authors confirmed the increased risk associated with this combination therapy (Rao et al., 2008). A meta-analysis comparing these agents with other glucose-lowering agents in T2D, reported an increased risk of stroke and overall mortality with sulphonylureas; however, their use was not associated with any significant difference in the incidence of major adverse cardiac events (MACEs) (including MI). Of note, none of the trials included in this meta-analysis was designed or powered to detect cardiovascular events (Azoulay and Suissa, 2017). Conversely, another recent study, evaluating second- or third-generation sulphonylureas, showed no negative effect with regard of all-cause mortality, cardiac mortality, MI or stroke in patients receiving these molecules (Varvaki Rados et al., 2016).

### Effects on Platelets

#### Preclinical Studies

Several *in vitro* studies have shown significant inhibition of ADP-induced platelet aggregation and a marked decrease in platelets adhesiveness with sulphonylureas (Klaff et al., 1981; Satoh et al., 1994). These anti-platelet effects might be mediated by the free radical scavenging ability of this class of agents. For the gliclazide molecule, this property relates to the unique presence of an amino-azabicyclo-octane ring, whereas, the platelet actions of glimepiride and glibenclamide seem to be linked to their influence on arachidonic acid metabolism (Violi et al., 1982; Satoh et al., 1994). Moreover, inhibitory effects of sulphonylureas on cyclooxygenase and lipoxygenase pathways have been reported (Satoh et al., 1994).

Klaff et al. investigated the effects of gliclazide and glyburide on platelets of diabetic patients, showing reduced aggregation in response to epinephrine and collagen compared with patients not receiving these agents (Klaff et al., 1981). Violi et al. reported an *in vitro* reduced activation in the thromboxane metabolic pathway after one-month treatment with gliclazide in a small cohort of T2D patients with abnormal platelet function (Violi et al., 1982). Other authors confirmed these findings with glyburide; this drug induced a significant suppression of phospholipase C's thrombin-induced activation with subsequent inhibition of platelet aggregation (Wada et al., 1999). Finally, glibenclamide has been demonstrated to inhibit platelet aggregation in murine platelets (Ting and Khasawneh, 2010).

#### Clinical Studies

The effect of glyburide on platelet aggregation was early investigated in a small clinical study enrolling 31 diabetic patients; a rapid (60 min) and significant decline in platelet aggregation after 5 mg glyburide ingestion compared to baseline was observed (de Bellis et al., 1984). Of note, gliclazide may be more effective in attenuating platelet aggregation than other sulphonylureas (Jennings et al., 1992; Konya et al., 2010). In particular Konya et al. demonstrated that T2D patients that switched from glibenclamide to gliclazide, titrating the dosage to reach the same glycemic control as the previous treatment (glibenclamide), exhibited a reduction in serotonin-induce platelet aggregates formation compared to that on glibenclamide treatment (Konya et al., 2010) (Table 1).

#### Interaction with Anti-Platelet Drugs

A recent study investigated the impact of concomitant use of sulphonylureas and clopidogrel on platelet function in a population of T2D patients undergoing PCI on DAPT (Harmsze et al., 2011). Sulphonylureas seemed to be associated with decreased platelet inhibition by clopidogrel quantified using ADP-induced light transmittance aggregometry. Of note, a 2.2-fold increased risk of high on-clopidogrel platelet reactivity was observed in diabetic patients treated with these anti-diabetic agents (Harmsze et al., 2011) (Table 2). These findings are probably due to the competition for isoenzyme CYP2C9 between sulfonylureas and clopidogrel. Interestingly, no drug–drug interactions due to competition for CYP2C9 between sulfonylureas and prasugrel or ticagrelor has been found. Conversely, the hypoglycemic effect of sulphonylureas seems to be potentiated if aspirin is co-administered; in fact, salicylates have been demonstrated to enhance sulphonylureas’ action by increasing insulin secretion. Moreover, aspirin may also displace sulphonylureas from its protein-binding site, enhancing their action (Krentz et al., 1994).

## Thiazolidinediones

Thiazolidinediones (TZDs) (pioglitazone, rosiglitazone and troglitazone) act as agonists of the peroxisome proliferator-activated receptor gamma (PPAR-γ), a pleiotropic nuclear receptor and transcription factor most highly expressed in adipose tissue and in macrophages, bone, liver and pancreatic beta cells among other tissues (Yau et al., 2013). Thus, TZDs regulate adipogenesis and fatty acid oxidation and promote adipose tissue ability to store lipids, decreasing circulating fatty acids and improving insulin resistance, whereas they decrease lipotoxicity and increase insulin sensitivity and glucose uptake in muscle (Boden et al., 2005).

### Cardiovascular Outcomes

Several randomized trials showed a potential for cardiovascular benefit with TDZs (Table 1). One of the largest trials, the PROspective Pioglitazone Clinical Trial In MacroVascular Events (PROactive) revealed a reduced incidence of the composite endpoint of death from any cause, nonfatal stroke or MI in T2D patients receiving pioglitazone compared with placebo in addition to other glucose-lowering drugs (Dormandy et al., 2005). However, these results have been counterbalanced to some extent by more frequent episodes of HF with pioglitazone (Dormandy et al., 2005). Similar data were reported from the Insulin Resistance Intervention after Stroke (IRIS) trial comparing pioglitazone vs. placebo in patients with insulin resistance and a recent stroke or transient ischemic attack (Viscoli et al., 2014). Patients assigned to TDZs reported a lower percentage of stroke or MI at 5 years compared with control subjects. Finally, a meta-analysis of major randomized controlled trials enrolling patients with stroke and abnormal glucose metabolism, showed that adding pioglitazone to standard therapy was associated with a 32% risk reduction of recurrent stroke and a 25% risk reduction of MACEs (DeFronzo et al., 2011).

Oppositely, another agent of this class, rosiglitazone, has been associated with an increased risk of cardiovascular events in several meta-analyses (Nissen SE et al., 2007; Selvin et al., 2008). The Rosiglitazone Evaluated for CV Outcomes in Oral Agent Combination Therapy for Type 2 Diabetes (RECORD) study was the only prospective trial to directly assess CV outcomes in patients treated with rosiglitazone in addition to metformin or sulphonylureas (Home et al., 2007; Home et al., 2009). The trial demonstrated no increase in the incidence of MI or cardiovascular death with this agent. However, the rate of HF hospitalizations was doubled in the rosiglitazone group compared with the control group (Home et al., 2009).

### Effects on Platelets

#### Preclinical Studies

In animal experiments, Sprague-Dawley rats treated with pioglitazone for 7–10 days showed delayed arterial thrombus formation by reduced ADP- and arachidonic acid-induced platelet aggregation (Li et al., 2005). Similarly, pioglitazone treatment for 1 week reduced soluble P-selectin and P-selectin platelet expression in an insulin-resistant, obesity-prone KK mouse model (Bodary et al., 2005). Ishizuka et al. demonstrated that troglitazone and vitamin E, but not pioglitazone, have potent inhibitory effects on platelet aggregation via suppression of the thrombin-induced activation of phosphoinositide signaling *in vitro* (Ishizuka et al., 1998).

#### Clinical Studies

In clinical studies, pioglitazone administered for 3 weeks in obese women showed a significant reduction in platelet activation assessed by urinary TXB metabolite excretion (Basili et al., 2006). Rosiglitazone has also been proved to reduce E-selectin (Hetzel et al., 2005), sCD40L and vWillebrand factor levels (Sidhu et al., 2003), procoagulant mediators released by activated platelets. Moreover, both rosiglitazone and pioglitazone demonstrated to reduce plasminogen activator inhibitor-1 (PAI-1) levels when administered in combination with glimepiride (Derosa et al., 2005). Despite these positive results, a sub-analysis of The Fixed Combination of Pioglitazone and Metformin Improves Biomarkers of Platelet Function and Chronic Inflammation in Type 2 Diabetes Patients (PIOfix) study, a randomized, double-blind study comparing the effects of pioglitazone plus metformin with metformin plus glimepiride on diabetic patients, reported slight but not significant improvements in platelet function markers such as 11-dhTXB2 levels in pioglitazone group, whereas a clear benefit was observed in E-selectin and vWillebrand factor concentrations (Schöndorf et al., 2011). Furthermore, Chokesuwattanaskul et al. reported that administration of 15 mg of pioglitazone daily for 7 days did not reduce platelet aggregation with ADP, epinephrine, collagen or arachidonic acid pathways in healthy, non-diabetic volunteers (Chokesuwattanaskul et al., 2010) (Table 1).

#### Interaction With Anti-Platelet Drugs

Mongan et al. (2012) investigated the potential platelet inhibitory effect of pioglitazone alone and in combination with aspirin. Platelet function was evaluated 90 and 180 min after ingestion of a single 30 mg dose of pioglitazone in both diabetic and normal subjects on aspirin. Importantly, a significant potentiating effect on platelet inhibition of pioglitazone in addition to aspirin was observed, and the percentage of patients with aspirin resistance, defined as an arachidonic acid-induced aggregation ≥20% despite aspirin, decreased from 63 to 28% after the addition of pioglitazone. Moreover, pioglitazone resulted in a significant decrease in 11-dhTXB2 release. Nevertheless, another small, randomized study, aiming to assess the impact of pioglitazone on clopidogrel-mediated P2Y12 inhibitory effects in T2D patients observed no benefit coming from this association. A 14-days treatment with pioglitazone 30 mg daily was not associated with any enhancement in clopidogrel antiplatelet activity assessed by light transmittance aggregometry and VerifyNow system compared with placebo (Suryadevara et al., 2012). On the other hand, a recent pharmacodynamics study reported that clopidogrel may increase the exposure to pioglitazone effects by inhibiting its CYP2C8-mediated biotransformation; in fact, one of the clopidogrel metabolites, clopidogrel acyl-beta-d-glucuronide, has been found to be a strong time-dependent inhibitor of CYP2C8 in humans and it may possibly overexpose patients to the effects of this anti-diabetic agent (Itkonen et al., 2016) (Table 2).

## Acarbose

Acarbose reduces postprandial hyperglycemia by inhibiting intestinal and salivary α-glucosidase; moreover, it acts with a non-competitive inhibition of pancreatic α-amylase (Hanefeld et al., 2008). Acarbose increases serum levels of Glucagon Like Peptide-1 (GLP-1) by regulating the reabsorption of bile acid or by stimulating L cells directly with the unabsorbed carbohydrates to achieve glycemic control (Zheng et al., 2013).

### Cardiovascular Outcomes

The effects of acarbose on cardiovascular outcomes were investigated in the Stop Non-Insulin-Dependent Diabetes Mellitus (STOP-NIDDM) trial (Chiasson et al., 2003), recruiting patients with impaired glucose tolerance randomly allocated to acarbose or placebo over a mean follow-up of 3.3 years. In this study, acarbose treatment was associated with a 49% risk reduction in the development of MACEs. Moreover, a meta-analysis of longer-term clinical trials of acarbose in patients with T2D confirmed the lower risk for cardiovascular events with this agent, most notably for MI (Hanefeld et al., 2004; Hanefeld et al., 2004a) (Table 1).

On the other hand, other trials failed to demonstrate cardiovascular benefits for this agent, such as the acarbose Cardiovascular Evaluation (ACE) trial, enrolling Chinese patients with established CVD and impaired glucose tolerance. In this study, acarbose treatment did not reduce the incidence of cardiovascular events during a 5-years treatment period (Holman et al., 2017). Notably, the absence of cardiovascular benefit in this trial compared with the STOP-NIDDM study might reflect the lower dose of acarbose used, the difference in the ethnic group, or the more aggressive secondary cardiovascular prevention measures being recommended when the ACE trial took place.

### Effects on Platelets

#### Preclinical Studies

In a rat animal model, Schafer et al. reported that chronic treatment with acarbose significantly reduced platelet activation due to acute hyperglycemia after oral administration of sucrose (Schäfer et al., 2004). Thus, the co-administration of acarbose, in addition to a sucrose load, reduced glucose absorption and attenuated platelet-bound fibrinogen and platelet surface-expression of P-selectin and glycoprotein 53 (Schäfer et al., 2004).

#### Clinical Studies

Santilli et al. evaluated the effects of acarbose on markers of lipid peroxidation and platelet activation (8-iso-PG F2α, 11-dhTXB2, plasma CD40 ligand and P-selectin) in T2D patients; 20-weeks acarbose treatment induced a time-dependent reduction in markers of oxidative stress and platelet activation that followed a progressive improvement of postprandial glucose fluctuations and long-term glycemic control (Santilli et al., 2010). Similar findings were reported also by Shimazu et al. (2009), demonstrating that acarbose significantly decreased platelet derived micro-particles and soluble P-selectin in patients with T2D. Furthermore, acarbose reduced *in vivo* platelet-monocyte aggregates formation in diabetic patients already at 60 min compared to untreated patients (Kaplar et al., 2001) (Table 1). No studies directly designed to analyze the interaction between acarbose and anti-platelets drugs have been published (Table 2).

## Dipeptidyl Peptidase-4 Inhibitors

Dipeptidyl peptidase-4 inhibitors (DPP4i) (alogliptin, linagliptin, vildagliptin, saxagliptin, sitagliptin) suppress the breakdown of incretin hormones GLP-1 and glucose-dependent insulinotropic peptide, achieving glycemic control. DPP4i showed beneficial effects on blood pressure, postprandial lipemia, inflammatory markers, oxidative stress and endothelial function in patients with T2D (Scheen, 2013). Moreover, they exhibit enzymatic activity against dozens of peptide hormones and chemokines with roles in vascular pathophysiology, inflammation, stem cell homing and cell survival (Mulvihill and Drucker, 2014).

### Cardiovascular Outcomes

Several randomized clinical trials have investigated the cardiovascular benefit of DPP4i with conflicting results (Table 1). However, most of those were *non-inferiority* studies, thus mainly aiming to demonstrate the safety rather than the effectiveness of these agents over standard glucose-lowering therapies.

In the Saxagliptin Assessment of Vascular Outcomes Recorded in Patients with Diabetes Mellitus (SAVOR-TIMI 53) and Examination of Cardiovascular Outcomes with Alogliptin vs. Standard of Care (EXAMINE) trials, DPP4i did not appear to increase the risk of MACEs (Scirica et al., 2013; White et al., 2013). Notwithstanding, both patients receiving saxagliptin and alogliptin experienced an increased incidence of HF admission compared with those randomized to placebo (HR 1.27, 95% CI 1.07–1.51, and HR 1.07, 95% CI 0.79–1.46, respectively). In light of these results, the Food and Drug Administration issued an updating warning over the cardiovascular safety of saxagliptin and alogliptin, to make patients and physicians aware of a potential increased risk of heart failure (*FDA Drug Safety Communication: FDA adds warnings about heart failure risk to labels of type 2 diabetes medicines containing saxagliptin and alogliptin* 2016)*.* Differently, the Trial Evaluating Cardiovascular Outcomes with Sitagliptin (TECOS) reported similar occurrence of cardiac events and HF hospitalizations in patients with T2D and CVD treated with sitagliptin and placebo (Green et al., 2015; Sharma et al., 2017).

No additional clinical benefit was observed with linagliptin compared with standard care in randomized clinical trials. Both the Cardiovascular Outcome Study of Linagliptin vs. Glimepiride in Patients With Type 2 Diabetes study (CAROLINA) (Marx et al., 2015), and, more recently, the CArdiovascular Safety and Renal Microvascular OutcomE with LINAgliptin in Patients With Type 2 Diabetes Mellitus at High Vascular Risk (CARMELINA) trial (Rosenstock et al., 2018; McGuire et al., 2019) did not report any difference in efficacy and safety outcomes between linagliptin and placebo.

Vildagliptin is the only DPP4i that lacks established or ongoing randomized controlled trials investigating cardiovascular outcomes; however, two large meta-analyses and a recent large european observational cohort study suggested that this agent was not associated with an increased risk of MACEs in a broad spectrum of populations (Mcinnes et al., 2015; Williams et al., 2017). Nevertheless, a small, randomized trial evaluating vildagliptin in patients with diabetes and HF with reduced ejection fraction, showed no increase in HF-related hospitalizations, but a harmful enlargement of left ventricular volumes of unknown cause with this drug (McMurray et al., 2018).

Considering the overall safety results from DPP4i randomized trials, these molecules must be used with caution in patients with increased risk of heart failure.

### Effects on Platelets

#### Preclinical Studies

Most of the DPP4i have been suggested to have anti-thrombotic effects. This is also related to their specific molecular structure: linagliptin, for example, contains a methylxanthine structure inhibiting phosphodiesterase and leading to increased intracellular cAMP levels. Thus, Steven et al. hypothesized that cAMP accumulation and subsequent phosphatase-A activation may represent one of the DPP4i anti-platelet mechanisms (Steven et al., 2017). In line with this, inhibition of DPP4 activity by diprotin A has been reported to induce platelet aggregation in human umbilical vein endothelial cells (Krijnen et al., 2012).

Additionally, DPP4 enzyme cleaves not only incretins, but also other substrates such as SDF-1α, peptide YY, and brain natriuretic peptide (Ussher and Drucker, 2012). Of these substrates, SDF-1α is the most potent chemokine known to induce cardiovascular protection by DPP4i, promoting stem cell repopulation and homing to ischemic tissues (Steven et al., 2017). Most of the DPP4i exhibited to suppress inflammatory and thrombogenic gene expression (Maeda et al., 2012; Matsubara et al., 2012; Birnbaum et al., 2016). Vildagliptin treatment has been shown to significantly reduce plasma fibrinogen and PAI-1 in an *in vivo* experiment (Dardik et al., 2003), whereas treatment of obese rats with saxagliptin decreased soluble levels of CD40 by over 10-fold (Mason et al., 2011). More recently, linagliptin was proven to significantly inhibit platelet aggregation by reducing platelet mitochondrial respiration and preserving cAMP-dependent phosphodiesterase. Moreover, this agent was also able to improve a carotid artery thrombosis model *in vivo* (Li et al., 2020).

#### Clinical Studies

Sitagliptin revealed to inhibit platelet aggregation in both healthy individuals and T2D patients. Fifty diabetic patients were treated with this agent for 3 months. After 1 and 3 months of DPP4i treatment, platelet aggregation, assessed *in vivo* by light transmission aggregometry, showed a significant reduction with sitagliptin (Table 1). The same findings were confirmed *in vitro*, using platelets from 10 healthy humans. Platelets pre-treated *in vitro* with 5 and 10 μg/ml of sitagliptin had a significant inhibition of thrombin-induced platelet aggregation (Gupta et al., 2012). Interestingly, the concentration-dependent antiplatelet activity of sitagliptin was attributed to the inhibitory effect on intracellular free calcium and tyrosine phosphorylation (Gupta et al., 2012).

## Sodium–Glucose Cotransporter-2 Inhibitors

Sodium–glucose cotransporter-2 inhibitors (SGLT-2i) (empagliflozin, canagliflozin, dapagliflozin) act by inactivating sodium glucose cotransporters located in S2 and S3 segments of renal proximal tubules, thus leading to a net loss of sodium and glucose through urine excretion and improving glycemic control via insulin-independent mechanisms (Cavallari and Maddaloni, 2019; Yaribeygi et al., 2019).

### Cardiovascular Outcomes

Large-scale randomized trials have demonstrated that SGLT-2i have a remarkable effect in reducing the incidence of MACEs in patients with T2D (Zelniker et al., 2019) (Table 1). Thus, the last European Society of Cardiology/European Association for the Study of Diabetes Guidelines recommend the use of this glucose-lowering class to lower the risk of HF hospitalization in patients with T2D and to reduce cardiovascular events in those at higher cardiovascular risk (Cosentino et al., 2020).

The Empagliflozin Cardiovascular Outcome Event Trial in Type 2 Diabetes Mellitus Patients–Removing Excess Glucose (EMPA-REG OUTCOME) was the first trial demonstrating an unexpected 34% risk reduction of cardiovascular death and HF admissions (35% risk reduction for the individual component of HF hospitalization) in patients treated with empagliflozin, corresponding to a number needed to treat to prevent one event of the composite outcome of 35 over 3 years (Zinman et al., 2015). Interestingly, even though only 10% of subjects in EMPA-REG trial had a pre-existing HF at baseline, the positive effects on HF hospitalizations and cardiovascular death were consistent in patients with or without previous HF (Fitchett et al., 2016). In accordance with the EMPA-REG OUTCOME, also the Canagliflozin Cardiovascular Assessment Study (CANVAS) trial observed a significant lower risk of CV death (22%) and HF hospitalization (33%) (Neal et al., 2017), whereas the Dapagliflozin Effect on Cardiovascular Events (DECLARE)-TIMI 58 trial reported a meaningful 17% reduction in the risk of the combined end point of cardiovascular death and HF hospitalizations with dapagliflozin (Kato et al., 2019).

A previous meta-analysis including more than 34,000 diabetic patients, of whom 60% with established cardiovascular disease, showed that SGLT-2i reduced the incidence of MACEs by 11%, as well as the incidence of cardiovascular death and HF hospitalization by 23% (Zelniker et al., 2019). Notably, SGLT-2i benefit emerged early after the initiation of the treatment and persisted on long-term follow-up (2–5 years). Moreover, if the reduction in MACEs occurrence was evident only in T2D patients with established CVD, the benefit in HF hospitalization was robust and consistent among the entire population, regardless of the previous presence of established atherosclerotic cardiovascular disease or a history of HF (Zelniker et al., 2019). These findings were confirmed by another recent meta-analysis containing results with ertuglifozin, another available SGLT2i (McGuire., et al., 2019). Indeed, SGLT2i are associated with a lower risk of MACEs, hospitalization for HF or cardiovascular death, and kidney outcomes, regardless of the presence or absence of pre-existing CVD.

Although the underlying mechanisms for the CV protection of SGLT-2i are not fully understood, a combination of several pathways are probably involved; the most studied mechanisms include the diuretic effect, reducing cardiac workload and myocardial oxygen consumption (Chilton et al., 2015; Gobel et al., 1978; Cavallari et al., 2020) and the beneficial effects of ketone bodies on the heart (Gobel et al., 1978; Chilton et al., 2015; Mudaliar et al., 2016; Sano et al., 2016; Pham and Chilton, 2017). Furthermore, recent investigations reported that empaglifozin is able to restore ion hemostasis in failing cardiac myocytes, reducing intracellular sodium overload by the inhibition of the sarcolemmal Na+/H+ exchanger (Bertero et al., 2018; Packer, 2019).

### Effects on Platelets

#### Preclinical Studies

Both direct and indirect antithrombotic effects of SGLT-2i have been suggested. In a recent study by Steven et al., 6-weeks treatment with empaglifozin significantly reduced inflammatory state, endothelial dysfunction and platelet hyperactivity/aggregation observed in the aorta of Zucker diabetic fatty rats (Steven et al., 2017). The same authors found that the up-regulation of mRNA expression for inducible nitric oxide synthase (eNOS) and P-selectin was prevented by SGLT-2i treatment (Steven et al., 2017). Spigoni et al. showed a reduced expression of ADP-induced platelet activation markers (CD62p and PAC1), assessed by flow-citometry, in platelets isolated from peripheral blood of healthy subjects and incubated with empaglifozin and dapaglifozin (Spigoni et al., 2020). Finally, a recent study investigating the direct effects of SGLT-2i on platelet aggregation, demonstrated that SGLT-2 protein is not expressed on human platelets. Moreover, in contrast to previous findings, the same authors reported that glifozins (canagliflozin, dapagliflozin, empagliflozin) produced only mild platelet inhibition, but their anti-thrombotic effects are strongly potentiated by NO and PGI_2_, thus with co-administration of sodium nitroprusside and iloprost (Lescano et al., 2020).

Furthermore, it has been observed that SGLT-2i can indirectly inhibit platelet function by reducing ROS generation. In this regard, a recent *in vitro* study showed that empaglifozin and dapaglifozin restored NO bioavailability in TNFα-stimulated human coronary arterial endothelial cells probably by inhibiting the generation of ROS (Wang et al., 1998; Smyth et al., 2017). Similarly, in another *ex vivo* study by Aroon and colleagues, empagliflozin suppressed advanced glycation end products, restored eNOS activation and reduced interstitial and periarterial NO stress (Aroor et al., 2018).

Nevertheless, volume depletion and hemoconcentration induced by SGLT-2i raised the question about a possible pro-thrombotic effect; concern that was disproved by several investigations. Indeed, some authors observed that the hematocrit rise triggered by these agents may promote oxygen delivery to the myocardium and reduce workload of the proximal renal tubules, improving tubule-interstitial hypoxia (Sano et al., 2016). Along this line, Santos-Gallego et al. recently investigated the effects of chronic (2 months) empagliflozin treatment on platelet aggregation in a porcine model after ischemic injury induced by coronary occlusion, failing to show enhanced platelet aggregation or thrombus kinetics alterations (Santos-Gallego et al., 2018) (Table 1).

#### Clinical Studies

No “ad hoc” study investigated the anti-platelet effects of SGLT-2i in diabetic patients. Indirect information can be deduced from large clinical trials. In 2018 Zheng and colleagues compared the efficacy of SGLT-2i, GLP-1 agonists, and DPP4i on mortality and cardiovascular end points using a network meta-analysis of 236 trials with 176 310 participants, finding that SGLT-2 inhibitors were associated with reduction in all MIs [HR 0.86 (95% CI 0.77–0.97)] and nonfatal MIs [HR 0.84 (95% CI 0.72–0.98)] compared with the control groups (Zheng et al., 2018). On the other hand, the supposed procoagulant activity of SGLT2 is due to the reduction of volemia was also excluded by several studies reporting no increase in venous thromboembolism with the use of these agents (Ueda et al., 2018). No studies specifically designed to investigate the interaction between SGLT-2i with anti-platelet drugs are available to date.

## GLP-1 Receptor Agonists

The incretin hormone GLP-1, mainly secreted from enteroendocrine cells after meal ingestion, exerts its insulin secretion and glucagon suppression effect in a glucose-dependent manner, upon binding to a specific GLP-1 Receptor (GLP-1R) (Sacks et al., 2018). This receptor represents the pharmacological target of GLP-1 receptor agonists (GLP-1RA). GLP-1 also inhibits beta-cell apoptosis, promotes beta-cell neogenesis, reduces circulating lipoproteins, delays gastric emptying and promotes satiety reducing food intake (Sacks et al., 2018).

### Cardiovascular Outcomes

Several randomized trials focused on GLP-1RA cardiovascular effects. A recent meta-analysis by Kristensen et al. (2019), including all the large, placebo-controlled, published randomized trials, summarized the evidence regarding GLP-1RA. The authors examined the incidence of MACEs (a composite outcome of CV death, non-fatal stroke and non-fatal MI) in the overall population but also in several specific subgroups based on patients’ characteristics (previous CVD, body mass index, age, baseline HbA1c), trial duration, treatment dosing interval and GLP1-RA structural homology. Seven trials with a total of 56,004 patients in primary (stable cardiovascular disease) and secondary cardiovascular prevention (cardiovascular risk factors) were included: Evaluation of Lixisenatide in Acute Coronary Syndrome (ELIXA), Liraglutide Effect and Action in Diabetes: Evaluation of Cardiovascular Outcome Results (LEADER), Trial to Evaluate Cardiovascular and Other Long-term Outcomes With Semaglutide in Subjects With Type 2 Diabetes (SUSTAIN- 6), Exenatide Study of Cardiovascular Event Lowering Trial (EXSCEL), Albiglutide and cardiovascular outcomes in patients with type 2 diabetes and cardiovascular disease (Harmony Outcomes), Dulaglutide and cardiovascular outcomes in type 2 diabetes (REWIND) and Trial Investigating the Cardiovascular Safety of Oral Semaglutide in Subjects With Type 2 Diabetes (PIONEER 6). In the pooled analysis, treatment with a GLP-1RA led to a 12% relative risk reduction in MACEs (HR 0.88 95% CI 0.82–0.94; *p* < 0.0001); when considered separately, the relative risk reduction for death from cardiovascular causes and for fatal and non-fatal stroke (12 and 16%, respectively) were also statically significant (*p* < 0.0001). Similarly, GLP-1RA treatment was associated with a significant reduction in the incidence of fatal or non-fatal MI (*p* = 0.043). In subgroup analyses, no statistical heterogeneity was reported between patients in primary or secondary prevention or when considering the baseline HbA1c; however, the authors found a potentially smaller clinical effect with drugs based on exendin-4 (exenatide and lixisenatide) compared with those more structurally similar to native GLP-1 (albiglutide, dulaglutide, liraglutide, and semaglutide). Moreover, since GLP1-RA benefit was consistent across subgroups based on age and kidney function, and older age and lower eGFR were associated with a higher rate of MACEs, it may be speculated that the positive effects of this class of glucose-lowering agents may be greater in these high-risk subgroups.

Importantly, this meta-analysis showed for the first time a reduction in HF admissions, more evident in two trials (Harmony Outcomes and LEADER), with the greatest reduction in MI, a common precursor of HF. These clinical findings may suggest that GLP-1RA have mainly an anti-atherothrombotic effect, with a less pronounced direct effect on HF than seen with SGLT-2i (Table 1).

### Effects on Platelets

#### Preclinical Studies

Several data suggest that the GLP1-RA may have a role in reducing platelet aggregation and thrombus formation (Table 1). Indeed, vascular smooth muscle cells appear to express the GLP-1 receptor which seems to exhibit a number of positive actions on the vascular endothelium (Almutairi et al., 2019). Moreover, recently, the presence of GLP-1R was also confirmed in platelets (Barale et al., 2017; Cameron-Vendrig et al., 2016; Marso, Bain, et al., 2016; Steven et al., 2017).

In a recent *in vitro* study, Cameron-Vendrig et al. demonstrated that incubation with exenatide elicited a cAMP response in human megakaryocyte cell line, leading to a significant inhibition of thrombin-, ADP-, and collagen-induced platelet aggregation (Cameron-Vendrig et al., 2016). This GLP-1RA was also able to reduce thrombus formation in *ex vivo* perfusion chambers using human and mouse whole blood and in a mouse artery model. According to this study, the anti-thrombotic effects of exenatide were mediated by a cAMP-induced phosphatase A activation, with subsequent enhancement of eNOS activity (Cameron-Vendrig et al., 2016). Similar data were reported in another experimental study by Steven et al. (2017), where platelet GLP-1R activation by liraglutide significantly reduced endotoxemia-induced microvascular thrombosis. Specifically, *in vitro* experiments on human platelets revealed a dose-dependent inhibitory effect of liraglutide on platelet activity in response to ADP and thrombin (Steven, Jurk, et al., 2017). Furthermore, other studies reported that the anti-platelet effects of GLP-1 metabolites and analogues could be related to their ability to decrease oxidative stress by improving intracellular antioxidant defenses and decreasing ROS production through GLP-1 receptor-dependent and independent pathways (Oeseburg et al., 2010; Ceriello et al., 2013).

#### Clinical Studies

Barale et al. (2017) investigated the effects of a 15-min incubation with the native form GLP-1 (7–36), its degradation product GLP-1 (9–36) and the GLP1-RA liraglutide in platelets from 72 healthy volunteers. They observed that all these agonists significantly attenuated platelet aggregation by inducing an increase in the NO bioavailability, by stimulating cGMP production and enhancing the extent of phosphorylation of vasodilator-stimulated phosphoprotein (VASP)-ser239, and by reducing the activation of phosphatidylinositol 3-kinase (PI3-K)/Akt and mitogen-activated protein kinase/extracellular-signal-regulated kinase (MAPK/ERK)-2 pathways probably through a cGMP-dependent protein kinase (PKG)-dependent mechanism. Furthermore, a significant decrease in ROS production was observed with circulating GLP-1 metabolites and liraglutide (Barale et al., 2017). Of note, in this study, GLP-1 metabolites and its analogues were demonstrated to act on platelet function independently from their receptor. In fact, the authors showed that when platelets were preincubated with exendin, a specific GLP-1R antagonist, the platelet inhibitory effects were maintained using GLP-1 (9–39), GLP-1 (7–36) and liraglutide. Conversely, more recently, the same authors (Barale et al., 2019) did not find any anti-thrombotic properties of GLP-1RA in subjects with primary hypercholesterolemia. In fact, GLP-1 (7–36), GLP-1 (9–36) and liraglutide all failed to enhance the anti-aggregating effects of exogenous NO and to reduce ROS production in platelets from patients with primary hypercholesterolemia compared with normocholesterolemic controls. Moreover, a lipid-lowering treatment with simvastatin for three months did not restore platelet sensitivity to GLP-1 effects. A possible explanation for these results could imply the same target of GLP-1 and simvastatin. In fact, both drugs seem to share, at least in part, inhibition of platelet NADPH oxidase-derived ROS formation, so the authors supposed that platelet exposure to GLP-1 after a treatment with simvastatin was no longer able to further modify resistance to GLP-1 related peptides.

In addition, Kahal et al. (2015) investigated anti-platelet effects of liraglutide in obese women with or without polycystic ovarian syndrome. They found a significant inhibition in basal platelet P-selectin expression after 6-months treatment with liraglutide.

No studies specifically designed to find interactions between GLP-1RA with anti-platelet drugs are available to date.

## Insulin Therapy

Indications for exogenous insulin therapy in patients with T2D include acute illness or surgery, pregnancy, glucose toxicity and contraindications to or failure to achieve goals with oral antidiabetic medications (Anon, 2020). Exogenous insulin is available as synthetic human insulin and insulin analogues, the latter being generally preferred. Insulins are typically classified based on their pharmacokinetic and pharmacodynamic (i.e., duration of action, absorption), as fast-acting (includes rapid-acting and short-acting insulins), intermediate-acting, and long-acting insulins.

### Cardiovascular Outcomes

In experimental models, insulin has both pro- and anti-atherogenic actions (Potenza et al., 2009), suggesting a mixed effect on CVD. However, data available from randomized trials consistently indicate an absence of cardiovascular safety issues with insulin therapy. In the UKPDS trial, the incidence of cardiovascular events in the insulin group was not different from those observed with sulfonylureas or in the control group on conventional therapy (Turner, 1998). However, in the long-term post-trial follow-up, authors showed that patients randomized to insulin therapy had a significant lower cardiovascular morbidity and mortality, possibly due to the long-term glycemic control improvement obtained with intensified insulin therapy (Gerstein et al., 2008). Similar results emerge from the Diabetes Mellitus, Insulin Glucose Infusion in Acute Myocardial Infarction (DIGAMI) Study, which demonstrated a significant 25% reduction in mortality (at 3.4 years of follow-up) among patients with diabetes and MI who underwent an initial 24h glucose-insulin infusion followed by an intensified insulin therapy (Malmberg, 1997). However, other CVD outcome trials failed to show any effect (beneficial or detrimental) of insulin therapy on CVD morbidity or mortality (Gerstein et al., 2012), confirming the cardiovascular safety of insulin treatment in T2D patients. In this regards, it is worth noting that in the Intensive Diabetes Treatment and Cardiovascular Outcomes in T1D Study (DCCT/EDIC) cohort, intensive diabetes therapy was associated with a modest reduction in all-cause mortality rate when compared with conventional therapy (Orchard et al., 2015), showing long-term beneficial effects on CVD incidence persisting for up to 30 years (Nathan, 2014).

The UKPDS findings and the potential cardioprotective effects on insulin among people with new-onset T2D were also tested in a large and long-term trial, the Outcome Reduction with an Initial Glargine Intervention (ORIGIN) study. In this trial, subjects were randomized to receive insulin glargine or standard of care on the basis of local guidelines (The ORIGIN trial investigators, 2012). There were two co-primary outcomes: the first included a composite MACEs outcome (non-fatal MI, non-fatal stroke or death from cardiovascular causes) and the second one any of these events plus a revascularization procedure or hospitalization for HF. At the end of the study (median follow-up 6.2 years) the incidence of both co-primary outcomes did not differ significantly between treatment groups. The authors suggested that the insulin cardiovascular benefits reported in previous large trials might have been mediated by improvements in glycemic control rather than direct cardiovascular effects.

## Effects on Platelets

The majority of studies on this topic combine *preclinical and clinical research*, which will be discussed in this section.

Physiologically, insulin has potent inhibitory effects on platelet hyperactivity, promoting NO production by activating eNOS through PI3K-dependent pathway and endothelin-1 secretion via MAPK-dependent pathway (Dimmeler et al., 1999; Mather et al., 2001). However this effect seems to be blunted in T2D. In fact, platelets of T2D patients have decreased sensitivity to insulin, which may lead to increased platelet reactivity and a higher risk of atherothrombotic events among insulin-treated subjects (Ferreira et al., 2006).

Spectre et al. investigated meal-induced platelet activation by comparing platelet P-selectin expression and fibrinogen binding, with or without stimulation with the thromboxane analog U46619 or ADP, following premeal injections of placebo or insulin aspart (Spectre et al., 2012). The standardized meal enhanced U46619-induced platelet P-selectin expression by 23% after placebo and it was more than doubled after premeal insulin. No changes in fibrinogen binding were found after meal intake with placebo, instead it was increased by 50–60% after premeal insulin (Spectre et al., 2012). These data suggest that postprandial hyperinsulinemia, rather than postprandial hyperglycemia, causes platelet hyperactivity mediated via pathways stimulated by TXB and ADP but not collagen in patients with T2D.

Few years later, the same authors, found no difference in the preprandial platelet activation between T1D and T2D patients; conversely, platelet-leukocyte aggregates were higher in T1D. After the standardized meal, platelet P-selectin expression, fibrinogen binding and platelet-leukocyte aggregates formation were approximately doubled in patients with T2D but unchanged in T1D. Thus, in the same study, 5 T1D volunteers were re-evaluated after their regular premeal insulin treatment, finding that, when premeal insulin was administered, platelet activation was increased. These findings confirmed the previous authors’ hypothesis that T1D patients, unable to secrete insulin, may have no platelet activation after the meal when no insulin is administered despite very high postprandial glucose levels, so postprandial platelet activation in diabetic patients may be more related to insulin rather than postprandial hyperglycemia (Spectre et al., 2016).

Interestingly, some data are available regarding the effect on platelets exerted by different type of insulin. Russo et al. showed that after 60 min of incubation of human platelets with insulin aspart or human regular insulin, the anti-aggregating effect was greater with insulin aspart than with human regular insulin (*p* = 0.027) (Russo et al., 2002).

However, the relationship between insulin therapy and platelet function remains controversial. The strongest evidence about the impact of insulin therapy on platelet activity appears to be more related to hyper or hypoglycemic events rather than to a direct effect on platelet function. On that note, insulin therapy in T2D is usually prescribed to patients with poor glucose control. Hyperglycemia enhances platelet activation and high-shear stress–induced activation, leading to arterial thrombosis, partly due to acute enhancement of the circulating levels of von Willebrand factor (Gresele et al., 2003). Furthermore, chronic hyperglycemia has been associated with a twofold rise in thrombin-antithrombin complexes and soluble tissue factor (Stegenga et al., 2006), nonenzymatic glycation of platelet glycoproteins, changes in their conformation and membrane lipid structure alterations (Angiolillo et al., 2006).

On the other hand, hypoglycemia, which is the main adverse effect of insulin therapy, also appears to be associated with platelet activation. A recent randomized trial showed that in healthy men acute hypoglycemia impairs fibrinolytic balance, increases platelet activation and coagulation biomarkers and reduces NO-mediated endothelial function (Joy et al., 2015). Additionally, other authors observed that in T1D subjects, hypoglycemic events result in activation of prothrombotic, proinflammatory and proatherogenic mechanisms (Gogitidze Joy et al., 2010).

In summary, chronic hyperglycemia as well as the higher risk of hypoglycemic events observed with insulin treatment appears to be more relevant that insulin physiological inhibition of platelet hyperactivity.

## Conclusion

Enhanced thrombosis mediates most serious complications associated with T2D such as MI and stroke, with a significant impact on survival. Thus, patients with T2D warrant careful attention of cardiologists and usually undergo more aggressive and longer anti-platelet regimens compared with non-diabetics.

Importantly, in the last years, a significant revolution in the management of diabetic patients has begun with the introduction of new glucose-lowering agents that have been demonstrated to meaningfully reduce the incidence of MACEs. In this review, we summarized the main cardiovascular effects of these agents, focusing on their anti-platelet properties potentially preventing thrombosis. Underlying mechanisms involve improved glycemic control, but also increased nitric oxide bioavailability, reduced oxidative stress and, for certain molecules, a direct inhibition of platelet activation and aggregation. Further investigations are needed to evaluate how much these anti-thrombotic abilities may contribute to the overall clinical benefit observed in randomized trials. Moreover, potential synergism with antiplatelet agents should be verified in *ad hoc* studies.

Surely, this evidence on old and new glucose-lowering agents introduces a paradigm change in the clinical management of T2D since we could potentially prevent thrombosis in such patients through an optimal use of anti-platelet but also glucose-lowering agents. Hence, cardiologists should be able to carefully manage these drugs in order to provide an appropriate therapy tailored to patients’ glycometabolic and thrombotic risk.

## Abbreviations

T2D, type 2 diabetes; T1D, type 1 diabetes; CVD, cardiovascular disease; MI, myocardial infarction; HF, heart failure; ROS, reactive oxygen species; PG, prostaglandin; ADP, adenosine diphosphate; PGI2, prostacyclin; NO, nitric oxide; PCI, percutaneous coronary intervention; TXB, thromboxane; DAPT, dual antiplatelet therapy; MACE, major adverse cardiac events; TZDs, Thiazolidinediones; PAI-1, Plasminogen activator inhibitor-1; GLP-1, Glucagon like peptide-1; DPP4i, Dipeptidyl peptidase-4 inhibitors; SGLT-2i, Sodium–glucose cotransporter 2 inhibitors; NOS, nitric oxide synthase; GLP-1RA, GLP-1 receptor agonists.
